# Unequal Behaviour between Hydrolysable Functions of Nirmatrelvir under Stress Conditions: Structural and Theoretical Approaches in Support of Preformulation Studies

**DOI:** 10.3390/pharmaceutics14081720

**Published:** 2022-08-17

**Authors:** Philippe-Henri Secretan, Maxime Annereau, Willy Kini-Matondo, Bastien Prost, Jade Prudhomme, Lina Bournane, Muriel Paul, Najet Yagoubi, Hassane Sadou-Yayé, Bernard Do

**Affiliations:** 1 Matériaux et Santé, Université Paris-Saclay, 92296 Châtenay-Malabry, France; 2 Clinical Pharmacy Department, Gustave Roussy Cancer Campus, 114 Rue Edouard Vaillant, 94800 Villejuif, France; 3 Department of Pharmacy, Henri Mondor Hospital, AP-HP, 94010 Creteil, France; 4 UMS-IPSIT-SAMM, Ingénierie et Plateformes au Service de l’Innovation Thérapeutique, Université Paris-Saclay, 92296 Châtenay-Malabry, France; 5 EpidermE, Université Paris Est Creteil, 94010 Creteil, France; 6 Department of Pharmacy, Pitié-Salpêtrière Hospital, AP-HP, 75013 Paris, France

**Keywords:** selective hydrolysis, ab initio calculations, DFT, mass spectrometry, degradation products, preformulation studies

## Abstract

Nirmatrelvir is an antiviral drug approved for the treatment of COVID-19. The available dosage form consists of tablets marketed under the brand name PAXLOVID^®^. Although knowledge of nirmatrelvir’s intrinsic stability may be useful for any potential development of other pharmaceutical forms, no data regarding this matter is available to date. Preliminary forced degradation studies have shown that the molecule is stable under oxidative and photolytic conditions, while hydrolytic conditions, both acidic and basic, have proven deleterious. Indeed, the molecule presents a priori several functions that can undergo hydrolysis, i.e., three amide moieties and a nitrile function. However, considering the degradation products formed under forced conditions and which were detected and identified by LC-UV-HRMS^n^, the hydrolysis process leading to their formation is selective since it involved only 2 of the 4 hydrolysable functions of the molecule. Ab initio studies based on density functional theory (DFT) have helped better understand these reactivity differences in aqueous media. Some hydrolyzable functions of nirmatrelvir differ from others in terms of electrostatic potential and Fukui functions, and this seems to correlate with the forced degradation outcomes.

## 1. Introduction

Nirmatrelvir (Paxlovid^®^, (1R,2S,5S)-*N*-[(1S)-1-cyano-2-[(3S)-2-oxopyrrolidin-3-yl]ethyl]-3-[(2S)-3,3-dimethyl-2-[(2,2,2-trifluoroacetyl)amino]butanoyl]-6,6-dimethyl-3-azabicyclo [3.1.0]hexane-2-carboxamide) is an antiviral drug approved for the treatment of COVID-19 [1].

Currently, nirmatrelvir exists only in tablet form, but it is very likely that for reasons such as facilitating its administration in pediatrics or geriatrics, liquid oral formulations will be needed or developed in the future. However, to the best of our knowledge, the literature is still lacking on the stability of nirmatrelvir in solution, where the degradation mechanisms and the products formed are missing. Furthermore, a thorough literature survey on compounds with similar structures such as, for instance, lufotrelvir, a drug containing similar groups to nirmatrelvir and interacting covalently with the same residue of the SARS-CoV2 3CL viral protease [2], did not allow us to find data that may enable us to anticipate the degradation pattern of nirmatrelvir.

One way of gaining rapid access to this type of knowledge is to study its behavior under stress conditions by characterizing in a non-targeted manner, for each condition, the products resulting from the degradation of the parent molecule, but within a limit of no more than approximately 15% degradation [3,4]. Applying this scheme, we quickly concluded that nirmatrelvir is a very stable molecule in solution under oxidative, photolytic or thermolytic conditions (data not provided).

It was under hydrolytic (acidic and basic) conditions that nirmatrelvir degraded. However, this is not surprising given the number of hydrolysable functions [5,6,7,8] that the molecule features. Unexpectedly, despite the exposure time of the active substance in the solution, these different functions did not act in the same way in the sense that some remained insensitive to acidic or basic pH while others were rapidly attacked.

In an attempt to rationalize this unexpected experimental result, a study to understand the theoretical reactivity of nirmatrelvir was performed with the help of density functional theory (DFT). The DFT approach can be used to determine the local reactivity properties of low molecular weight organic molecules [9,10] and has already been used for drugs containing amide functions [5,6].

The structural characterization of the hydrolysis products and DFT reactivity studies to elucidate the potential causes of this unequal behavior are presented in this article.

## 2. Materials and Methods

### 2.1. Reagents and Sample Preparation

Nirmatrelvir tablets (Paxlovid^®^) are marketed by Pfizer (New York, NY, USA). Analytical grade acetonitrile came from Sigma-Aldrich (St Quentin-Fallavier, France). Ultrapure water was produced by the Q-Pod Milli-Q system (Millipore, Molsheim, France). Hydrogen peroxide (H_2_O_2_) 30% *v*/*v* was supplied by Carlo Erba SDS (Val de Reuil, France).

A stock standard solution of nirmatrelvir was prepared by extracting nirmatrelvir from crushed tablets. Three tablets were crushed, the coating was removed by sieving, and nirmatrelvir was extracted from the remaining powder using pure methanol to get a final concentration of about 1 mg mL^−1^ of nirmatrelvir. Methanol was chosen as it is a solvent in which nirmatrelvir is freely soluble, whereas the excipients of the tablets (cellulose, microcrystalline, lactose monohydrate, croscarmellose sodium, hydrophobic colloidal silica, sodium stearyl fumarate) are sparingly soluble at best in methanol or ethanol. This suspension was then filtered, and the resulting solution was diluted with ultrapure water so as to obtain a final concentration of 300 µg mL^−1^ in every working solution depicted thereafter. The amount of nirmatrelvir in the extract was assessed by preparing a solution by dissolving the nirmatrelvir substance (Sellek chemicals, Purity: 99.82%) at 1 mg·mL^−1^. The yield was determined to be 99.3%.

Hydrolysis was studied at room temperature over a period of 1 week, using HCl 1 M (final pH: 1.0 ± 0.2) and NaOH 1 M (final pH: 13.0 ± 0.2). The chosen conditions were based on those typically used to investigate the degradation of drugs under acidic and basic conditions [3,4].

### 2.2. Instrumental

Chromatographic separations were achieved using a Dionex UltiMate 3000 HPLC system (Les Ulis, France) controlled by Chromeleon^®^ software version 6.80 SR11 (Dionex, Les Ulis, France). The selected column was a Phenomenex C18 (250 nm × 4.6 nm; 5 µm), maintained at 30 °C. The flow rate, the injection volume and the wavelength of detection were set at 1 mL min^−1^ 20 µL and 220 nm, respectively. Gradient mode combining 0.1% (*v*/*v*) formic acid added in both solvents: pure water (solvent A) and acetonitrile (solvent B) was used to separate and detect compounds over a wide range of polarities. The gradient program was set as follows: 0–2 min: 95% A; 2–30 min: 95 → 0% A; 30–35 min: 95% A. The flow-rate entering the mass spectrometer was decreased down to 0.3 mL min^−1^ using a 1/3 T split.

Mass spectra were acquired by using an LTQ-Orbitrap Velos Pro system (Thermo Fisher Scientific, Waltham, MA, USA). MS in negative ion mode was used, but MS signals in negative ion mode were poor for nirmatrelvir and its degradation products. Thus, analysis was carried out in positive ion mode (ESI^+^) as per the following conditions: the source voltage was set at 3.4 kV, the source and the capillary temperatures were fixed at 300 °C and 350 °C, respectively. Sheath gas and auxiliary gas nitrogen flows were set at 35 and 15 arbitrary units, respectively. S-lens was set at 60%. Normalized collision was set at 35 for high-resolution fragmentation studies. The mass range of 105–1200 amu was used for preliminary high resolution-mass spectrometry (HR-MS) studies and that of 50–700 amu, for HR-MS^n^ studies. The chosen full width at half maximum (measured at fifty percent of the maximum peak height) was 60,000 for all stages of HR-MS^n^. Instrumentation calibration was performed daily based on the recommendations of the manufacturer of the mass spectrometer and using the Pierce™ LTQ Velos ESI positive ion calibration solution (ThermoFisher, Waltham, MA, USA). The MS data were processed using Xcalibur^®^ software (version 2.2 SP 1.48, ThermoFisher, Waltham, MA, USA).

### 2.3. Computational Conditions

Jaguar [11] and Maestro (Maestro, Schrödinger, LLC, New York, NY, USA, 2019) programs of Schrödinger Suite 2019-4 have been used for the preparation and visualization of results of the studied molecule. The choice of nirmatrelvir 3D’s neutral conformer model for geometry optimization was based on the one published by Pubchem^®^ (https://pubchem.ncbi.nlm.nih.gov, accessed on 1 May 2022). For geometry optimization, a B3LYP exchange-correlation functional with the D3 a posteriori correction [12] has been chosen, together with the 6-311G++** basis set.

Molecular Electrostatic Potential (MEP), a descriptor providing information about the charge distribution that is useful for identifying molecular sites likely interacting with other molecules [10], was mapped as a function of electron density. Fukui f^−^ and f^+^ functions were also mapped as they, respectively, give information on the electro donating and withdrawing sites of the studied compound [11].

## 3. Results

LC-UV and LC-HR-MS analyses were first performed to assess the global reactivity of nirmatrelvir (Section 3.1). The detected degradation products were then characterized by LC-HRMS^n^ (3.2), which enabled us to propose a degradation pattern. The unexpected lack of reactivity under hydrolytic stress of some of the amid groups was investigated by use of DFT.

### 3.1. Degradation Products Detected under Hydrolytic Conditions

**Figure 1:**
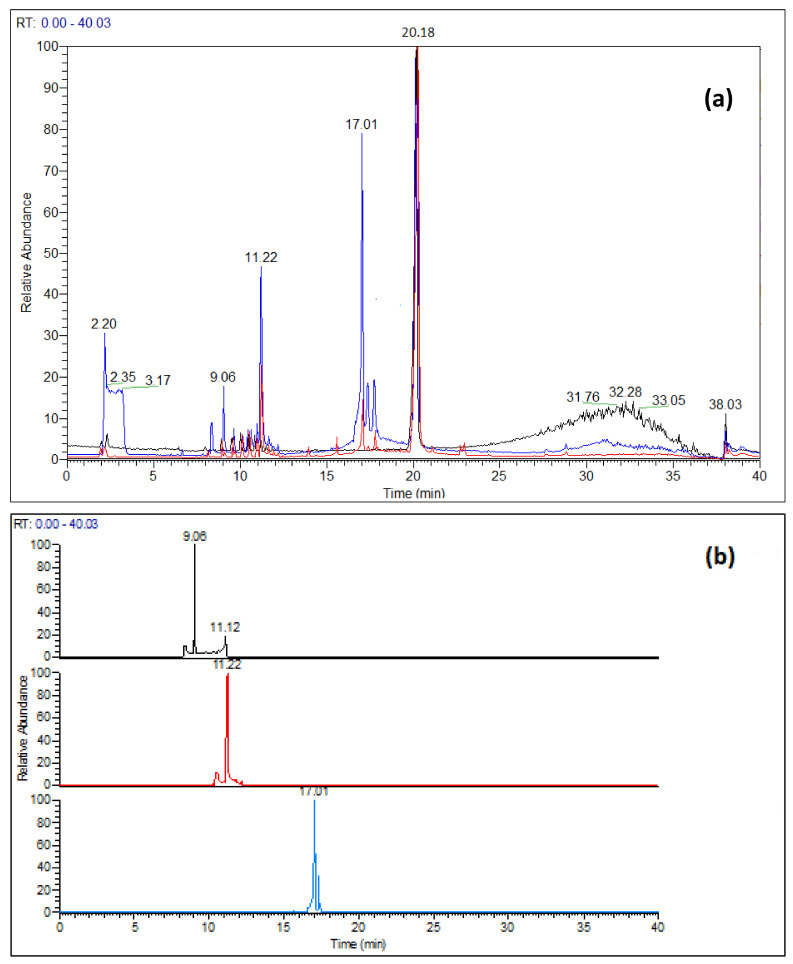
(**a**) LC-HR-MS full scan chromatograms of nirmatrelvir: standard solution (black), after 3 h at pH = 13 ± 0.2 (blue), after one week at pH = 1 ± 0.2 and at 40 °C (red). (**b**) Extracted ion chromatograms obtained from the full scan chromatogram recorder after 3 h at pH = 13 ± 0.2: black *m/z* [421.5*–*422.5] uma, red: *m/z* [403.5*–*404.5] uma, light blue: *m/z* [517.5*–*518.5] uma.

When nirmatrelvir was exposed to hydrolytic conditions, three degradation products were detected by UV detection (Figure S1) and mass spectrometry (Figure 1a,b), two of which are common to both acidic and basic conditions and a third was only detected under basic conditions. The three degradation products were named as a function of their monoisotopic mass. The resulting extracted ion chromatograms of protonated DP_421_ [DP_421_+H]^+^), DP_403_ [DP_403_+H]^+^) and DP_517_ [DP_517_+H]^+^) are showed in Figure 1b. DP_421_ (retention time = 9.06 min) is the least intensely detected DP in basic conditions and is undetected under acidic conditions.

### 3.2. Structural Characterization of the DPs

Structural characterization of the DPs relied on the in-depth study of the mass spectrum of nirmatrelvir and its DPs, with the aim to pinpoint characteristic fragmentation process. The proposed structures were then supported by proposing a process of formation.

#### 3.2.1. Protonated Nirmatrelvir’s Fragmentation Pattern

**Figure 2:**
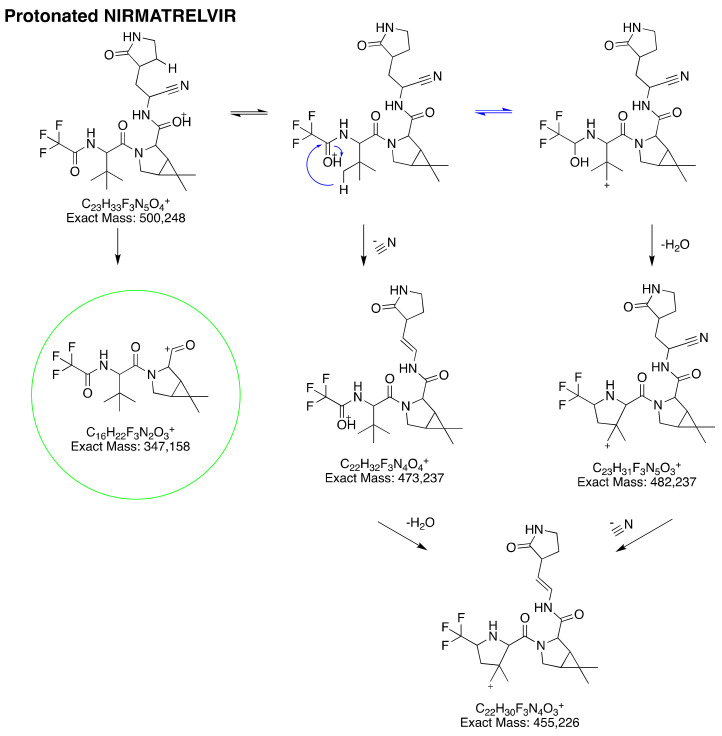
Proposed fragmentation pattern of protonated nirmatrelvir. The structure of the base peak-ion is circled in green.

The LC-HR-MS² mass spectrum of nirmatrelvir protonated ion (C_23_H_33_F_3_N_5_O_4_^+^) displays four intense ions with accurate masses of 482.237, 473.237, 455.227 and 347.158 uma (Figure S2), which correspond to the respective molecular formulas: C_23_H_31_F_3_N_5_O_3_^+^ C_22_H_32_F_3_N_4_O_4_^+^ C_22_H_30_F_3_N_4_O_3_^+^ and C_16_H_22_F_3_N_2_O_3_^+^. The first two ions (C_23_H_31_F_3_N_5_O_3_^+^ and C_22_H_32_F_3_N_4_O_4_^+^) match separate neutral loss of water and cyanide, whereas the third product ion (C_22_H_30_F_3_N_4_O_3_^+^) corresponds to the combination of the two processes (Figure 2). The base peak-ion, C_16_H_22_F_3_N_2_O_3_^+^ (accurate mass: 347.1579), circled in green in Figure 2, is characteristic of the 3,3-dimethyl-2-[(2,2,2-trifluoroacetyl)amino]butanoyl]-6,6-dimethyl-3-azabicyclo[3.1.0]hexane-2-carboxamide part of nirmatrelvir. These neutral losses and the base peak-ion have been very helpful for the structural elucidation of the DPs.

#### 3.2.2. Structural Elucidation of DP_517_

**Figure 3:**
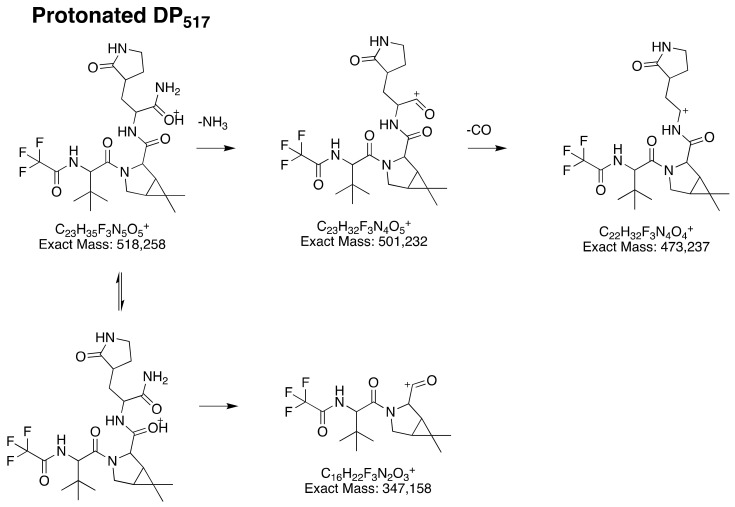
Proposed fragmentation pattern of protonated DP_517_ ([DP_517_+H]^+^).

Based on its accurate mass, protonated DP_517_ formula is consistent with C_23_H_35_F_3_N_5_O_5_^+^ and corresponds to nirmatrelvir hydration. LC-HRMS² and LC-HRMS^3^ mass spectra (Figures S3 and S4) show three ions intensely detected ions (501.232, 473.238 and 347.158 uma), which correspond to C_23_H_35_F_3_N_4_O_5_^+^, C_22_H_32_F_3_N_4_O_4_^+^ and C_16_H_22_F_3_N_2_O_3_^+^, respectively. Featuring the same molecular formula than the base peak-ion found for nirmatrelvir (which structure is circled in green in Figure 2), it can be inferred that DP_517_ had kept this part of the molecule identical to that of nirmatrelvir. The other two ions (C_23_H_35_F_3_N_4_O_5_^+^ and C_22_H_32_F_3_N_4_O_4_^+^) are formed by successive neutral losses of ammoniac and carbon oxide, negligible phenomenon highlighted for nirmatrelvir. Therefore, the presence of these two ions, along with the absence of the cyanide loss strongly detected in the study of nirmatrelvir’s fragmentation pattern, have strongly suggested that the nitrile moiety be transformed in amide, giving rise to ammoniac and carbon oxide losses (Figure 3). As a result, DP_517_ corresponds to *N*-(1-amino-1-oxo-3-(2-oxopyrrolidin-3-yl)propan-2-yl)-3-(3,3-dimethyl-2-(2,2,2-trifluoroacetamido)butanoyl)-6,6-dimethyl-3-azabicyclo[3.1.0]hexane-2-carboxamide.

#### 3.2.3. Structural Elucidation of DP_403_

**Figure 4:**
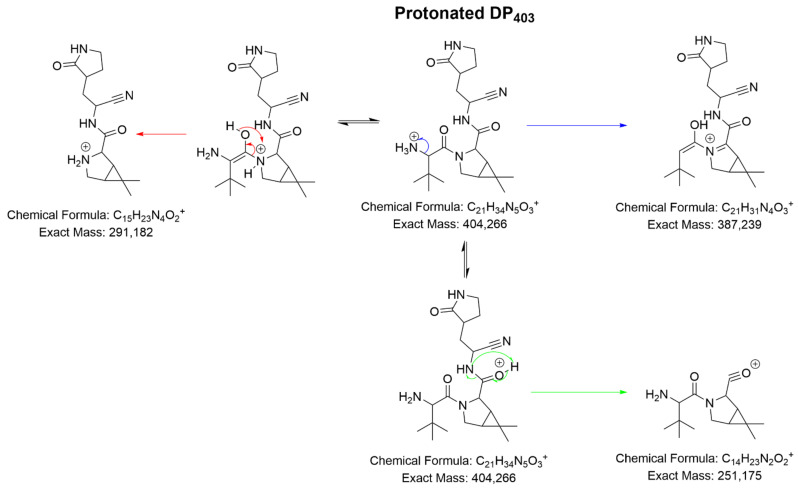
Proposed fragmentation pattern of protonated DP_403_ ([DP_403_+H]^+^).

The accurate mass of protonated DP_403_ ([DP_403_+H]^+^) is consistent with the formula C_21_H_34_N_5_O_3_^+^ and the structure presented in Figure 4, i.e., a product formed from the hydrolysis of the trifluoroacetamide radical. The LC-HRMS² studies (Figure S5) strongly support this claim given the structure of the ions identified and the comparative studies with the data presented for nirmatrelvir (Figure 2). Based on its structure, DP_403_ should be named 3-(2-amino-3,3-dimethylbutanoyl)-*N*-(1-cyano-2-(2-oxopyrrolidin-3-yl)ethyl)-6,6-dimethyl-3-azabicyclo[3.1.0]hexane-2-carboxamide.

#### 3.2.4. Structural Elucidation of DP_421_

**Figure 5:**
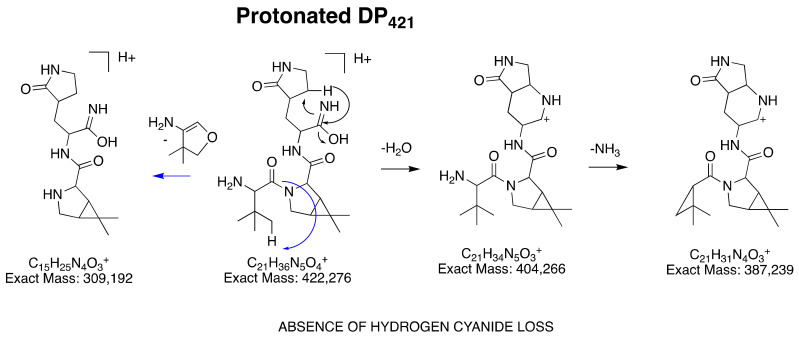
Proposed fragmentation pattern of protonated DP_421_ ([DP_421_+H]^+^).

With the C_21_H_36_N_5_O_4_^+^ formula, based on the accurate mass of its protonated ion, DP_421_ can unambiguously result from two processes, the one that led to DP_517_ and the one that led to DP_403_. The fragmentation pattern of protonated DP_42*1*_ ([DP_421_+H]^+^), based on LC-HRMS² studies (Figure S6), confirmed its structure (Figure 5). As a result, DP_421_ corresponds to *N*-(1-amino-1-oxo-3-(2-oxopyrrolidin-3-yl)propan-2-yl)-3-(2-amino-3,3-dimethylbutanoyl)-6,6-dimethyl-3-azabicyclo[3.1.0]hexane-2-carboxamide.

### 3.3. Main Degradation Process of Nirmatrelvir Characterized by Mass Spectrometry

The regions of nirmatrelvir that are basically prone to hydrolysis are the oxopyrrolidine ring, the nitrile group, the trifluoroacetamide radical, and the *N*-methyl acetamide moiety (see structure in Figure 2). Against all odds, from the structure of the previously identified degradation products detected under hydrolytic conditions, attacks only occurred on the nitrile group and the trifluoroacetamide radical, despite prolonged expositions to acidic or basic media.

The mechanisms of degradation of nirmatrelvir, thus highlighted, are described below.

#### 3.3.1. Nirmatrelvir Degradation under Basic Conditions

**Figure 6:**
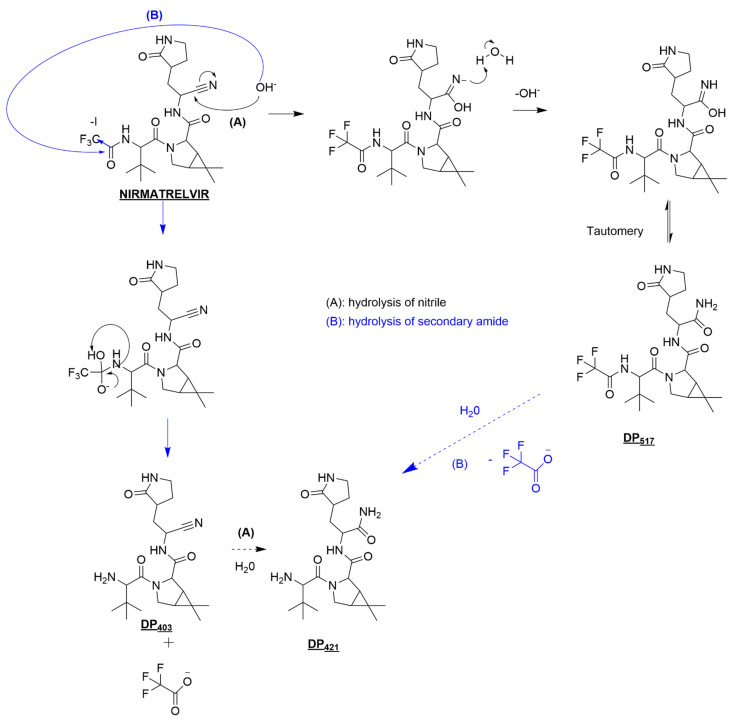
Proposed nirmatrelvir’s degradation pathways under basic conditions.

When exposed to basic conditions, the nitrile group of nirmatrelvir can be attacked as per path A proposed in Figure 6. Briefly, nucleophilic attack by hydroxide ions occurs on the carbon of the nitrile group, giving rise to an azanide intermediate, which then captures hydrogen from a water molecule. By tautomeric rearrangement, two forms of DP_517_ may coexist, amide or methanimidic acid derivative.

Hydroxide ions can also attack the carbonyl-carbone located in alpha of the -CF_3_ moiety and causes after rearrangement, the departure of trifluoroacetate along with the formation of DP_403_ (path B).

DP_421_, on the other hand, results from the involvement of these two pathways and this is moreover an element that confirms their sole involvement, in the absence of other hydrolysis products coming from attacks on other hydrolysable parts of the parent molecule.

#### 3.3.2. Nirmatrelvir Degradation under Acidic Conditions

**Figure 7:**
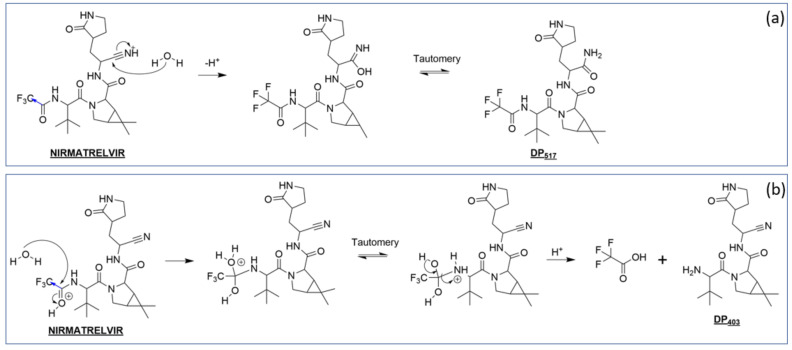
Proposed nirmatrelvir’s degradation pathways under acidic conditions leading to the formation of DP_517_ (**a**) and DP_403_ (**b**).

Degradation pathways taking place under acidic catalysis are proposed in Figure 7. Although the pathways leading to their formation are different, DP_517_ (inset (a)) and DP_404_ (inset (b)) are also formed under acidic conditions, demonstrating one more time that the attacks did indeed occur only on the same chemical groups. After protonation of the corresponding carbonyl group or of the nitrile function, in both cases, water addition occurs. Rearrangements that take place afterwards explain the onset of DP_517_ and DP_403._ In acidic media, the amide or methimidic acid functions are much less prone to hydration, explaining why DP_421_ was undetected.

### 3.4. Preferential Hydrolysis Sites of Nimatrelvir, an Investigation by DFT

Based on previous stress studies, only the nitrile group and the trifluoroacetamide radical are sensitive to hydrolytic conditions, whereas one would expect the other amide functions to undergo hydrolysis as well. To better understand this difference, some local reactivity properties of nirmatrelvir as a function of pH were studied by DFT.

#### 3.4.1. Reactivity of Nirmatrelvir to Acidic pH

**Figure 8:**
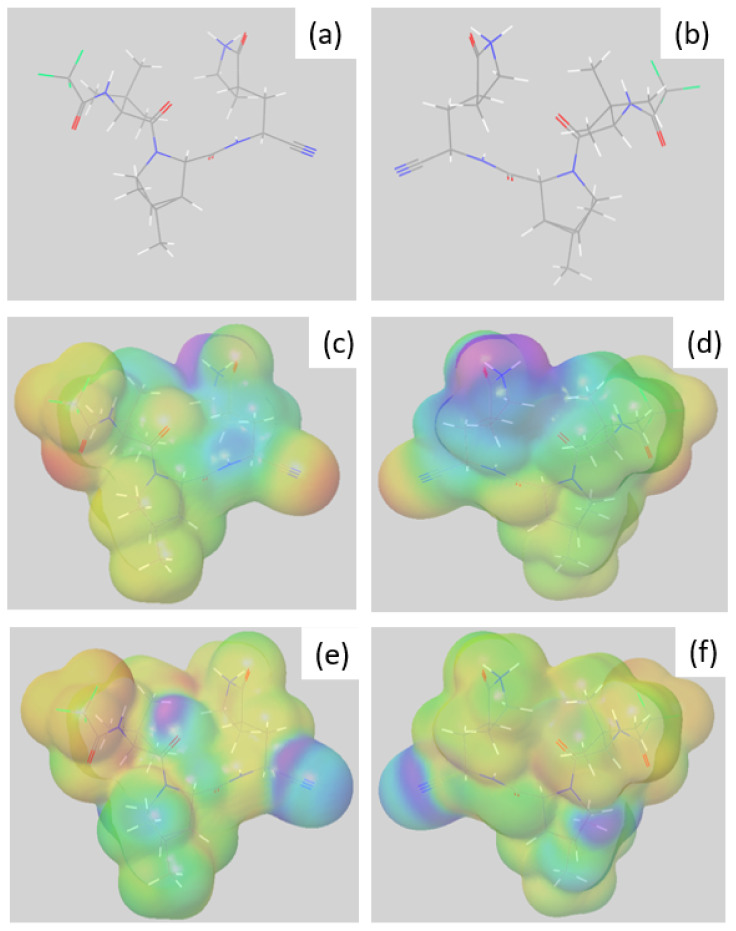
(**a**,**b**) Structure of optimized geometry of nirmatrelvir’s main conformer at pH = 1; (**c**,**d**) mapped electrostatic potential; and (**e**,**f**) mapped Fukui f^−^ function. Regions with the lowest and highest values are mapped in red and purple, respectively.

To assess the theorical reactivity of nirmatrelvir in acidic conditions (pH = 1), a first step is to obtain the optimized geometry of its predominant form in water and at this pH. In this condition, the nitrogen of the lactam moiety is amenable to gaining a hydrogen atom, leading to the optimized structure depicted in Figure 8a,b.

Then, based on the optimized structure, Molecular Electrostatic Potential (MEP) was mapped as a function of electron density as it provides a good basis for the identification of critical molecular sites in terms of local reactivity [13]. Indeed, MEP descriptor gives information about the charge distribution and is useful for identifying molecular sites likely interacting with other molecules. Fukui f^−^ function was also mapped, as they give information on the electro donating sites of the studied compound [14].

MEP surfaces (Figure 8c,d) at acidic pH showed that the lowest electrostatic potentials are specifically located on the nitrile group (mapped in red, Figure 8c,d) and to a lesser extent, the trifluoroacetamide radical (mapped in yellow and red, Figure 8c,d). A lower potential may indeed imply an ability to react more with other chemical entities around, in this case with H_2_O at first.

These results are partly in line with the isosurface obtained when mapping the Fukui function f^−^ (Figure 8e,f). Indeed, the region in purple and green in the figure are the one where atoms that are most likely to act as Lewis bases, confirming that the nitrile group is the region most prone to react.

Combining MEP and the Fukui function f^−^ results, it seems that the main regions of the parent molecule that can undergo acidic attacks are the nitrile function and the trifluoroacetamide radical, which is consistent with the results from the forced degradation studies carried out under acidic conditions (see Section 3.2.2).

#### 3.4.2. Reactivity of Nirmatrelvir to Basic pH

**Figure 9:**
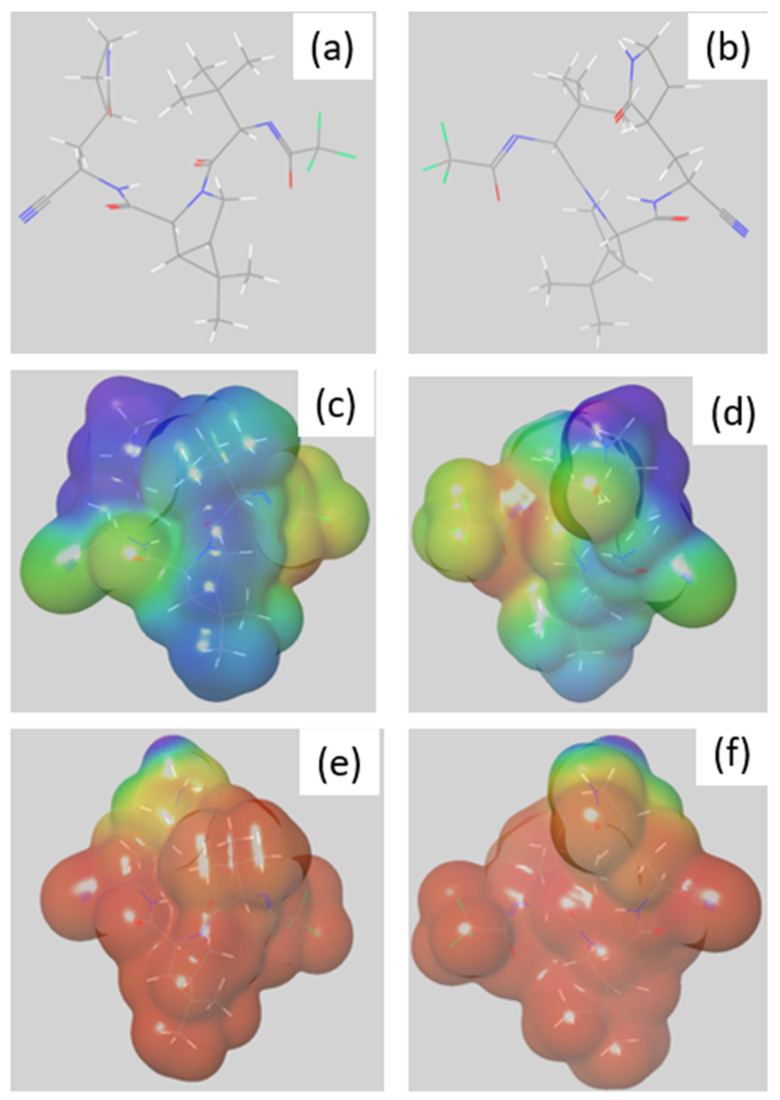
(**a**,**b**) Structure of optimized geometry of nirmatrelvir’s main conformer at pH = 13; (**c**,**d**) associated mapped electrostatic potential; and (**e**,**f**) mapped Fukui f ^+^ function. Regions with lowest and highest values are mapped in red and purple, respectively.

Under basic conditions, the Fukui function to investigate is f^+^ instead of f^−^ as it is more relevant to consider the electrophilic parts of the molecule. The optimized structure of the main form of nirmatrelvir under basic conditions and its associated MEP and f^+^ function are gathered in Figure 9.

Overall, these data are consistent with those presented in Section 3.4.1 and Section 3.3.1, proposing the same or even the only groups to be attacked under the stress conditions tested.

In addition to these results, this study also gave us a better understanding of why the oxopyrrolidine ring (purple, Figure 9c,d) did not open due to hydrolysis. Its f ^+^ function (in purple, Figure 9e,f) indeed indicates the part of the molecule with the lowest propensity to react under these hydrolytic conditions.

## 4. Conclusions

In the absence of data on the stability of nirmatrelvir in aqueous medium and under stress conditions, we have investigated this aspect, showing that the molecule is stable under different stress conditions, except under hydrolytic conditions. These results are encouraging in the sense that future or potential developments of liquid forms will not encounter any real difficulties except to try to optimize the pH in favor of stability in solution or suspension, something we did not explore in this study.

On the other hand, on a mechanistic level, we were able to highlight and explain reasons for a selective hydrolysis of certain hydrolysable functions among others of the molecule, thanks to the concordance of the experimental and theoretical results.

These fundamental data complement other data from pre-formulation studies, which are particularly useful for the development of other dosage forms when it comes to providing tailored responses to specific needs related to the administration of nirmatrelvir.

## Data Availability

Not applicable.

## Supplementary Materials

The following supporting information can be downloaded at: https://www.mdpi.com/article/10.3390/pharmaceutics14081720/s1, Figure S1: (a) LC-UV chromatograms of nirmatrelvir: standard solution (black), after 3 h at pH = 13 ± 0.2 (blue), after one week at pH = 1 ± 0.2 and at 40 °C (pink). (b) Detailed results of the chromatograms exposed in inset (a), Figure S2: LC-HRMS² mass spectrum of protonated nirmatrelvir, Figure S3: LC-HRMS² mass spectrum of protonated DP_517_, Figure S4: LC-HRMS3 mass spectrum of the ion of mass to charge ratio 501 obtained after fragmentation of protonated DP_517_, Figure S5: LC-HRMS² mass spectrum of protonated DP_403_, Figure S6: LC-HRMS² mass spectrum of protonated DP_421_.
