# Supplementary material for: Unequal Behaviour between Hydrolysable Functions of Nirmatrelvir under Stress Conditions: Structural and Theoretical Approaches in Support of Preformulation Studies
Source: Pharmaceutics. 2022 Aug 17;14(8):1720. doi: 10.3390/pharmaceutics14081720 (PMC9412384; doi:10.3390/pharmaceutics14081720)
Supplement: Supplementary file 1 [file pharmaceutics-14-01720-s001.zip › pharmaceutics-1824354-supplementary.pdf]

Supplementary material to :

Unequal behavior among hydrolyzable functions of nirmatrelvir under stress conditions in preformulation studies: structural and theoretical studies

Philippe-Henri Secretan[1,*], Maxime Annereau[1,2], Willy Kini Matondo[3], Bastien Prost[4], Jade Prudhomme[1], Lina Bournane[1], Muriel Paul[3,5], Najet Yagoubi[1], Hassane Sadou-Yayé[1,6], Bernard Do[1,3]

1 Matériaux et santé, Université Paris-Saclay, 92296, Châtenay-Malabry, France

2 Clinical Pharmacy Department, Gustave Roussy Cancer Campus, 114 rue Edouard Vaillant, 94800 Villejuif, France

3 Department of Pharmacy, Henri Mondor Hospital, AP-HP, 94010 Créteil, France

4 UMS-IPSIT-SAMM, Ingénierie et Plateformes au Service de l'Innovation Thérapeutique, Université Paris-Saclay, 92296, Châtenay-Malabry, France

5 EpidermE, Université Paris Est Creteil, 94010 Creteil, France

6 Department of Pharmacy, Pitié-Salpêtrière hospital, AP-HP, 75013 Paris, France

Table of contents

Figure S1: (a) LC-UV chromatograms of nirmatrelvir: standard solution (black), after 3 hours at pH=13 +/- 0.2 (blue), after one week at pH=1 +/- 0.2 and at 40°C(pink). (b) detailed results of the chromatograms exposed in inset (a)……………………………………………………………………………………………3

Figure S2: LC-HRMS^2 mass spectrum of protonated nirmatrelvir... 3

Figure S3: LC-HRMS^2 mass spectrum of protonated DP_{517}... 4

Figure S4: LC-HRMS^3 mass spectrum of the ion of mass to charge ratio 501 obtained after fragmentation of protonated DP_{517}... 4

Figure S5: LC-HRMS^2 mass spectrum of protonated DP_{403}... 5

Figure S6: LC-HRMS^2 mass spectrum of protonated DP_{421}... 5

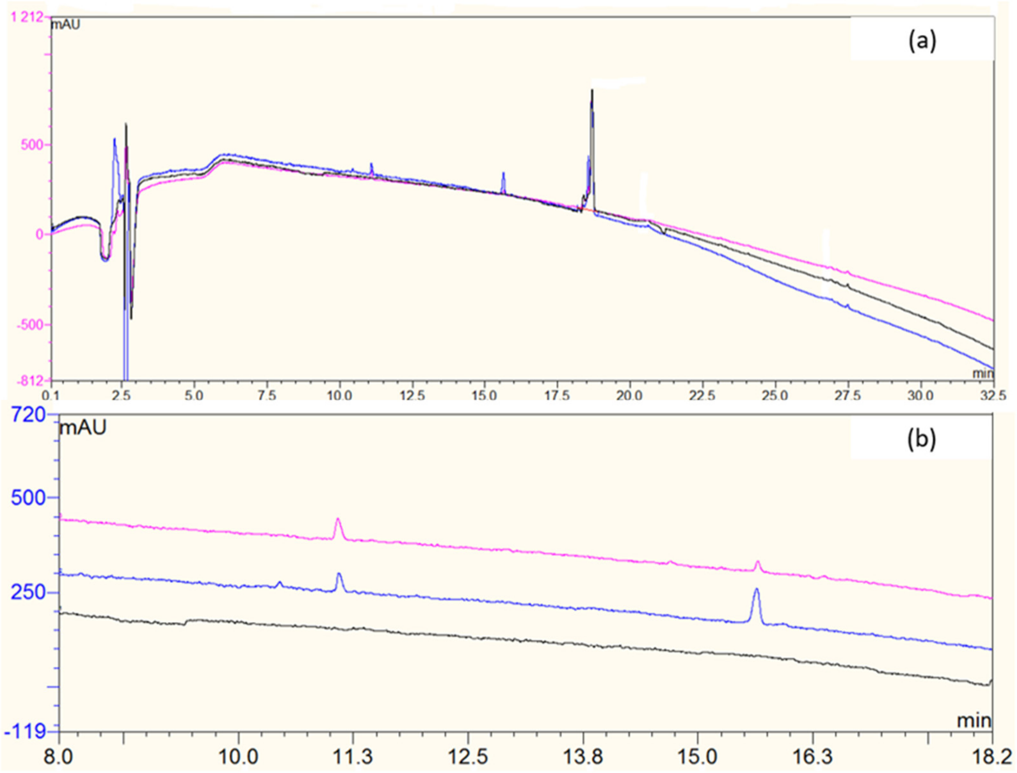

Figure S1: (a) LC-UV chromatograms of nirmatrelvir: standard solution (black), after 3 hours at pH=13 +/- 0.2 (blue), after one week at pH=1 +/- 0.2 and at 40°C(pink). (b) detailed results of the chromatograms exposed in inset (a).

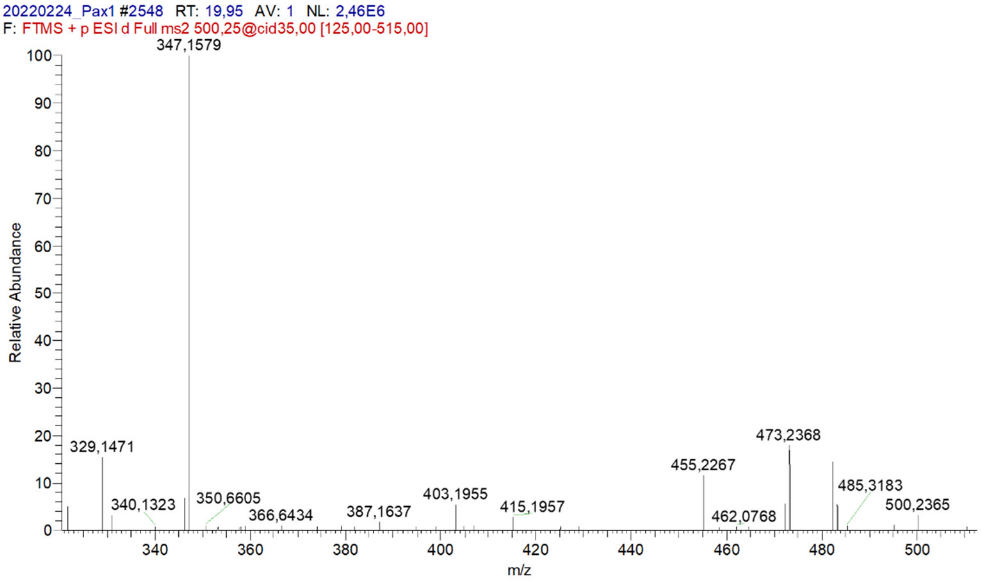

Figure S2: LC-HRMS^2 mass spectrum of protonated nirmatrelvir.

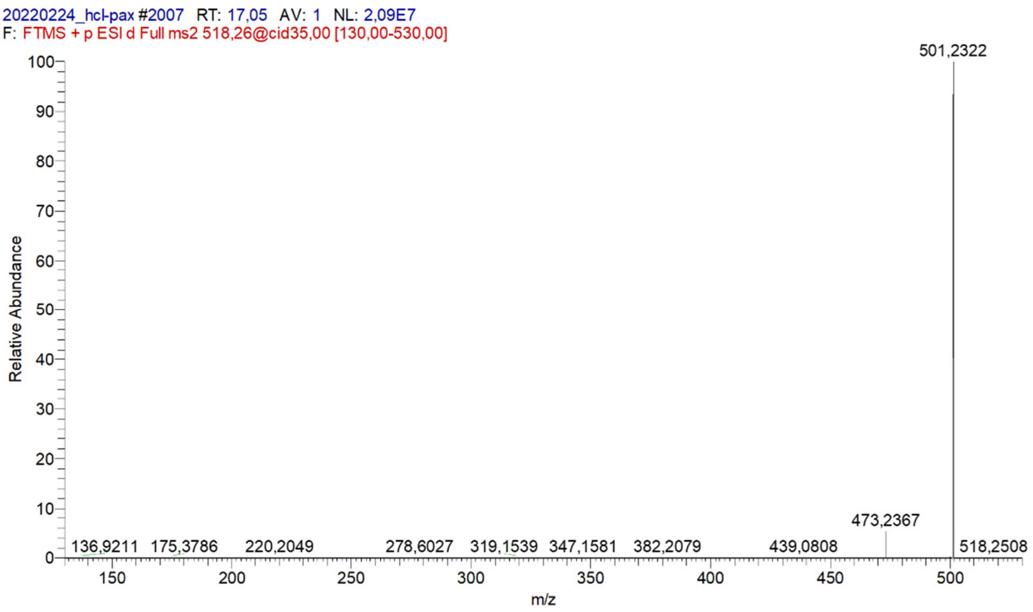

Figure S3: LC-HRMS^2 mass spectrum of protonated DP_{517}.

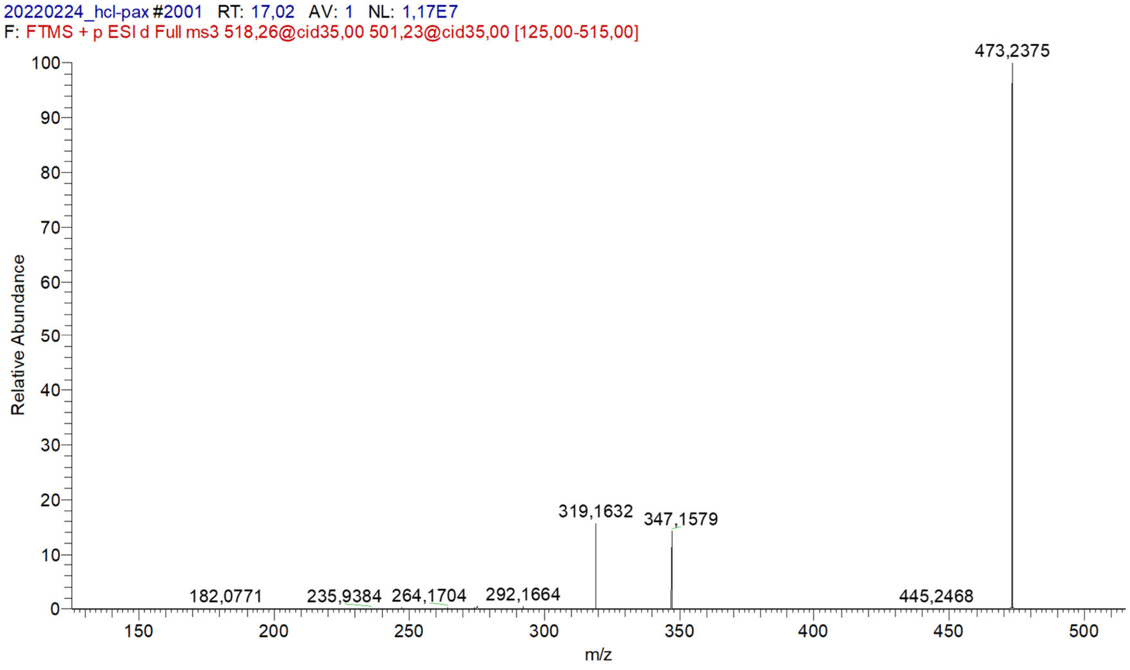

Figure S4: LC-HRMS^3 mass spectrum of the ion of mass to charge ratio 501 obtained after fragmentation of protonated DP_{517}.

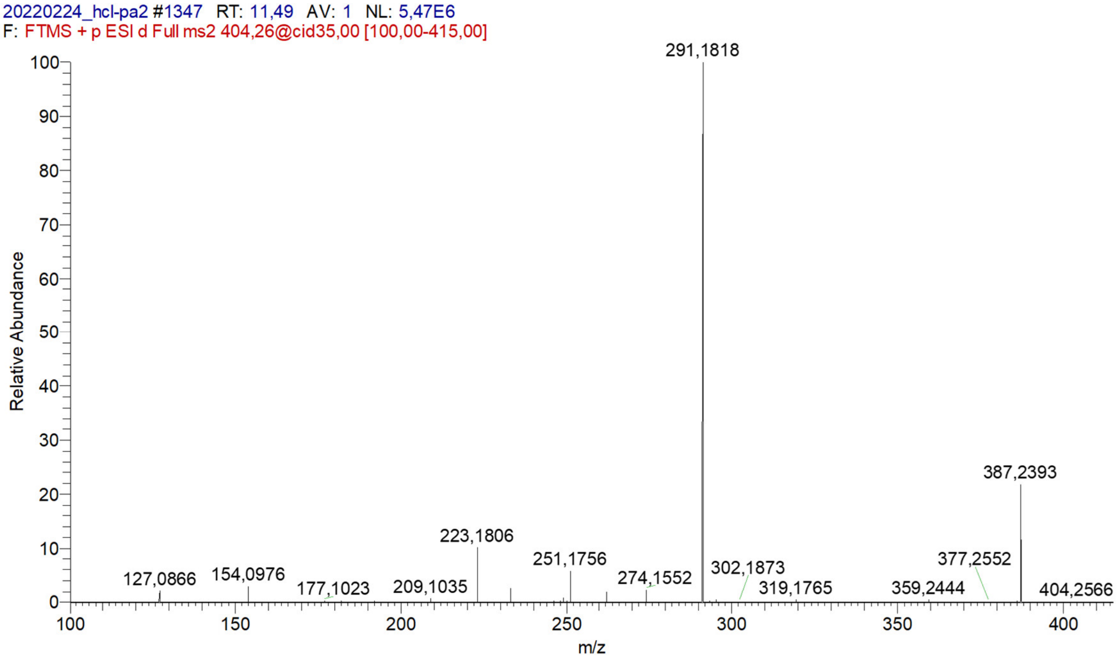

Figure S5: LC-HRMS^2 mass spectrum of protonated DP_{403}.

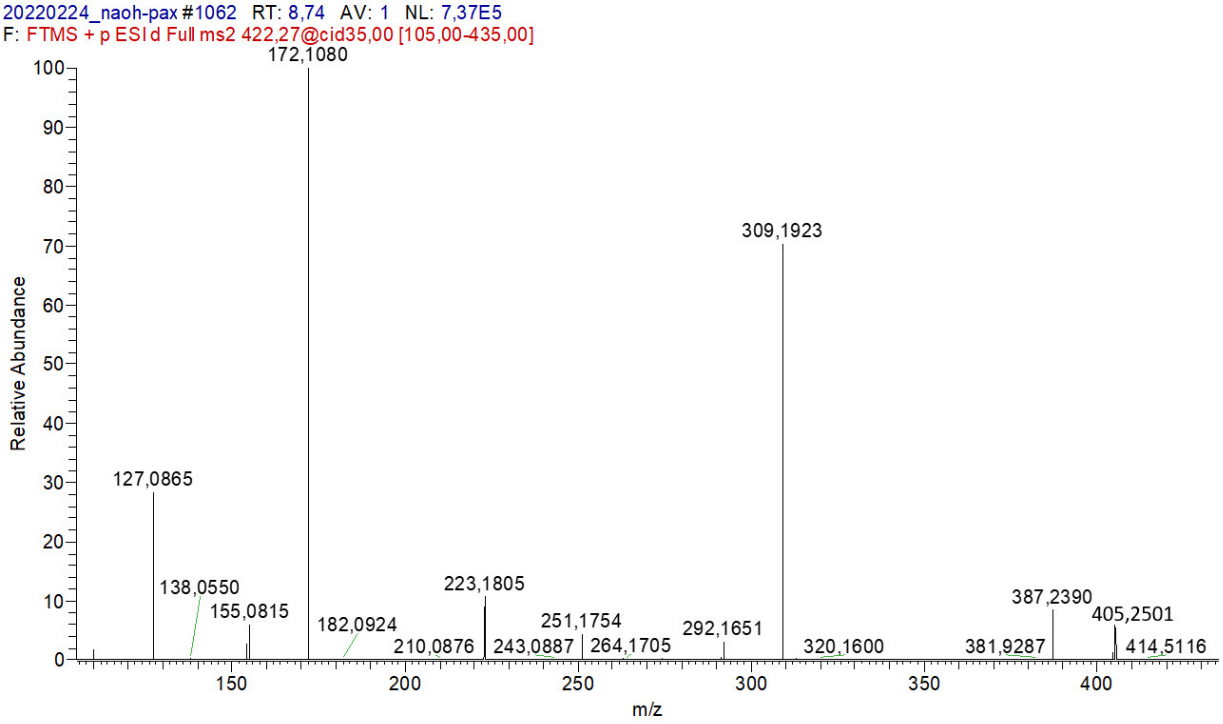

Figure S6: LC-HRMS^2 mass spectrum of protonated DP_{421}.
